# Prognostic Value of Qualitative Splenic [^18^F]FDG Uptake on Baseline PET/CT in Newly Diagnosed Diffuse Large B-Cell Lymphoma

**DOI:** 10.3390/cancers18030449

**Published:** 2026-01-30

**Authors:** Yunhwi Hwang, Sung Hwa Bae, Sang Jun Byun, Byungwook Choi

**Affiliations:** 1 Division of Hematology-Oncology, Department of Internal Medicine, Daegu Catholic University Medical Center, Daegu Catholic University School of Medicine, Daegu 42472, Republic of Korea; soprano37@naver.com (Y.H.); sunghwa@cu.ac.kr (S.H.B.); 2 Department of Radiation Oncology, Keimyung University School of Medicine, Daegu 42601, Republic of Korea; kryph@dsmc.or.kr; 3 Department of Nuclear Medicine, Daegu Catholic University Medical Center, Daegu Catholic University School of Medicine, Daegu 42472, Republic of Korea

**Keywords:** diffuse large B-cell lymphoma, [^18^F]FDG PET/CT, splenic uptake, prognostic factor, relapse-free survival, overall survival

## Abstract

**Background/Objectives**: Accurate risk stratification at diagnosis is crucial for the optimal management of diffuse large B-cell lymphoma (DLBCL). While Fluorine-18 fluorodeoxyglucose positron emission tomography/computed tomography ([^18^F]FDG PET/CT) is the standard imaging modality for staging, the prognostic value of non-tumoral uptake patterns remains under investigation. Here, we aimed to investigate the prognostic significance of qualitative splenic [^18^F]FDG uptake on baseline PET/CT in patients with newly diagnosed DLBCL, focusing on relapse-free survival (RFS) and overall survival (OS). **Methods**: This retrospective study included consecutive patients with newly diagnosed DLBCL in a Korean cohort who underwent baseline [^18^F]FDG PET/CT between December 2016 and August 2023. Qualitative splenic uptake was visually assessed on maximum intensity projection images. Associations between splenic uptake, prognostic indices, and clinicopathologic characteristics were evaluated. Survival outcomes and independent prognostic factors were analyzed using Kaplan–Meier methods and Cox proportional hazards regression models. **Results**: A total of 142 patients were analyzed (43 relapsed, 58 died). Positive splenic [^18^F]FDG uptake was observed in 72 patients and was significantly more frequent in patients who relapsed (*p* < 0.001). Positive splenic uptake was significantly associated with inferior RFS (*p* < 0.001) and OS (*p* = 0.010). For RFS, advanced Ann Arbor stage, ECOG performance status, and extranodal involvement were also significant factors. In multivariable analysis, positive splenic uptake remained an independent predictor of poorer RFS (hazard ratio 2.175, *p* = 0.043), along with advanced stage (hazard ratio 2.872, *p* = 0.004). **Conclusions**: Qualitative splenic [^18^F]FDG uptake on baseline PET/CT is associated with adverse clinical outcomes in patients with DLBCL and serves as an independent prognostic factor for RFS.

## Simple Summary

Diffuse large B-cell lymphoma is an aggressive blood cancer where predicting outcomes at diagnosis is vital for treatment planning. [^18^F]FDG positron emission tomography/computed tomography ([^18^F]FDG PET/CT) is routinely used to assess disease extent by detecting metabolic activity, yet visual features outside tumor sites remain underutilized. This study focused on the qualitative visual assessment of splenic [^18^F]FDG uptake on baseline scans, avoiding complex quantitative measurements. We found that patients showing visually increased splenic uptake at diagnosis were significantly more likely to experience relapse and had poorer survival outcomes. Importantly, this feature predicted relapse independent of established clinical risk factors. These findings suggest that a simple visual check of the spleen on routine [^18^F]FDG PET/CT provides valuable prognostic information and is easily applicable in daily clinical practice.

## 1. Introduction

Diffuse large B-cell lymphoma (DLBCL) is the most common aggressive subtype of non-Hodgkin lymphoma (NHL) and remains a major cause of lymphoma-related morbidity and mortality. In the United States, the American Cancer Society estimates that there will be 80,350 new NHL diagnoses and 19,390 NHL deaths in 2025, reflecting the continued population-level burden of lymphoma care [1]. Because DLBCL is the most common NHL subtype across geographic regions—accounting for approximately 25–30% of NHL in Western Europe and roughly one-third in North American White populations, with even higher proportions reported in parts of Latin America and Asia—it constitutes a substantial fraction of newly diagnosed NHL cases requiring prompt therapy [2]. In Korea, nationwide analyses of mature B-cell NHL have shown that DLBCL is the most frequently diagnosed subtype, accounting for approximately 41.9–48.4% of NHL cases in recent registry-based cohorts [3]. These epidemiologic patterns emphasize the clinical importance of identifying readily applicable prognostic markers at the time of diagnosis.

Despite therapeutic advances driven by rituximab-containing chemoimmunotherapy, clinical outcomes in patients with DLBCLs remain heterogeneous. A clinically meaningful proportion of patients develop primary refractory disease or relapse after first-line therapy, motivating continued efforts to refine baseline risk stratification. Contemporary prognostication still relies heavily on clinical models such as the International Prognostic Index (IPI), the Revised IPI (R-IPI), and the National Comprehensive Cancer Network-IPI (NCCN-IPI), which incorporate age, stage, serum lactate dehydrogenase (LDH), performance status, and extranodal involvement [4,5,6,7]. In a large pooled analysis, discrimination for overall survival was modest, with concordance indices of 0.632 for the NCCN-IPI, 0.626 for the IPI, and 0.590 for the R-IPI [7]. Although these indices are widely validated and remain clinically actionable, substantial within-group variability persists, indicating that clinical variables alone may not fully capture the disease biology or host–tumor interactions relevant to relapse and survival.

Given the high FDG avidity of aggressive B-cell lymphomas, fluorine-18 fluorodeoxyglucose positron emission tomography/computed tomography ([^18^F]FDG PET/CT) has become integral to DLBCL management. International lymphoma imaging guidance and the Lugano framework emphasize PET/CT for baseline staging and response assessment, recommending standardized visual interpretation using the Deauville five-point scale for routine reporting and clinical trial harmonization [8,9]. Baseline PET/CT contributes to accurate whole-body mapping of nodal and extranodal disease, can direct biopsy to the most metabolically active site, and supports subsequent therapy monitoring [9]. In parallel, multiple studies—particularly in large contemporary cohorts—have shown that baseline PET-derived tumor-burden metrics, most notably total metabolic tumor volume (and related glycolytic burden measures such as total lesion glycolysis), are associated with progression and survival outcomes in DLBCL, with reported hazard ratios (HR) for adverse events ranging from approximately 1.7 to 3.3 in patients with high metabolic burden [10,11,12]. However, while these parameters can improve prognostic stratification, they may require time-intensive segmentation, dedicated software, and institution-specific methodological choices, limiting routine adoption in some clinical environments.

The spleen is the largest secondary lymphoid organ and plays central roles in immune surveillance, lymphocyte trafficking, cytokine signaling, and clearance of circulating antigens. In hematologic malignancies, imaging evaluation of the spleen is clinically important because splenic involvement and splenic lesion morphology/metabolic activity can alter staging, treatment planning, and prognosis [13]. Splenic findings on cross-sectional imaging and PET/CT may reflect a spectrum ranging from direct lymphomatous involvement to reactive immune-metabolic activation. Prior radiologic literature has described the spectrum of splenic imaging patterns in hematologic malignancies and highlighted the value of PET/CT for detecting metabolically active splenic involvement compared with anatomic imaging alone [14].

Despite this biologic plausibility and routine visualization on baseline PET/CT, spleen-focused PET assessment has been less systematically evaluated as a prognostic tool in newly diagnosed DLBCL than tumor-burden metrics or response-adapted PET strategies. Recent work has examined splenic abnormalities on pretreatment PET/CT in DLBCL, including studies assessing focal splenic lesions and their association with progression-related outcomes [15]. Moreover, emerging analyses have explored whether uptake in “healthy tissues,” including the spleen, carries prognostic information in baseline DLBCL PET/CT datasets [16]. However, the evidence remains limited and heterogeneous in methods and endpoints. Importantly, it remains unclear whether a simple qualitative assessment of baseline splenic uptake adds incremental prognostic value when integrated with established clinical indices (IPI, R-IPI, NCCN-IPI) in newly diagnosed DLBCL. From a practical standpoint, diffuse splenic uptake is readily assessed on routine maximum intensity projection and fused PET/CT images without specialized segmentation. If splenic metabolic activity reflects clinically relevant tumor–host biology or subclinical dissemination, it may serve as a reproducible imaging biomarker that complements established clinical models while avoiding the operational complexity of volumetric tumor quantification.

Therefore, in this study, we sought to evaluate splenic [^18^F]FDG uptake on baseline PET/CT in patients with newly diagnosed DLBCL and to determine its association with relapse-free survival (RFS) and overall survival (OS). Specifically, we aimed to (i) characterize qualitative and quantitative splenic metabolic parameters at diagnosis; (ii) examine their relationships with clinicopathologic factors and established prognostic indices; and (iii) test whether incorporating splenic uptake into IPI-, R-IPI-, and NCCN-IPI-based models improves prognostic discrimination.

## 2. Materials and Methods

### 2.1. Patients

**Figure 1:**
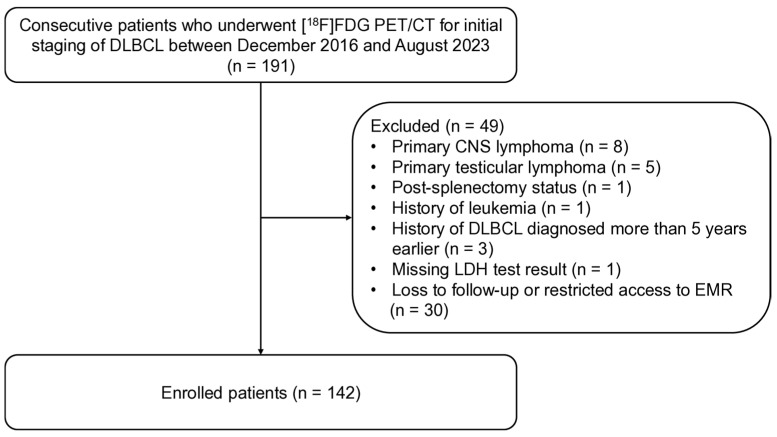
Flowchart of this study.

Patients who were newly diagnosed with DLBCL at our institution between December 2016 and August 2023 were retrospectively screened. The inclusion criteria were as follows: (1) histopathological confirmation of DLBCL and (2) availability of baseline [^18^F]FDG PET/CT performed at the time of initial diagnosis. The exclusion criteria were as follows: (1) primary central nervous system lymphoma or testicular lymphoma; (2) incomplete clinical data required for IPI calculation; (3) prior history of DLBCL or other hematologic disorders such as leukemia or multiple myeloma; (4) inability to evaluate splenic uptake; (5) baseline [^18^F]FDG PET/CT performed more than 1 month before or after the initial diagnosis; and (6) patients who did not receive any treatment or were lost to follow-up after diagnosis. The flowchart of the patient selection process is shown in Figure 1. All patients included in this study were of East Asian ethnicity (Korean). Our institutional review board approved this study, and the need for obtaining written informed consent from the participants was waived due to its retrospective design (IRB No. 2025-11-026).

### 2.2. Data Collection and Survival Endpoints

Patient demographics, clinical characteristics, and laboratory data were obtained from the hospital’s electronic medical records and the Picture Archiving and Communication System. Follow-up information was retrieved solely from the electronic medical record system, and the observation period was completed in August 2025. The principal survival outcomes of interest were RFS and OS. RFS was defined as the duration from the date of histopathologic confirmation of DLBCL to the first evidence of relapse, either local or distant; patients without relapse were censored at the time of last follow-up or death. OS was defined as the interval from diagnosis to death from any cause or last follow-up. Relapse events were determined by clinicians using imaging studies and/or pathological confirmation.

### 2.3. [^18^F]FDG PET/CT Acquisition

All [^18^F]FDG PET/CT examinations were performed using a Discovery IQ PET/CT system (GE Healthcare, Milwaukee, WI, USA). Prior to tracer administration, patients fasted for at least 6 h, and blood glucose levels were confirmed to be below 150 mg/dL. A [^18^F]FDG dose of 4.0 MBq/kg was injected intravenously. Approximately 60 min after tracer injection, PET/CT images covering the region from the skull base to the proximal thighs were obtained. Low-dose, non-contrast CT scans were performed for attenuation correction and anatomical localization using the following parameters: tube voltage, 120 kVp; smart mA modulation, 30–80 mAs depending on body weight; rotation time, 0.5 s; helical thickness, 3.75 mm; pitch, 0.938; table speed, 18.75 mm/rotation; and matrix, 512 × 512. Subsequently, PET emission data were acquired from the cerebellum to the proximal thigh, with a matrix size of 256 × 256 and an acquisition time of 3 min per bed position. PET data were reconstructed using the Q.Clear algorithm provided by the manufacturer, which applies a block-sequential regularized expectation maximization method with a penalization factor (β = 400).

### 2.4. Qualitative and Quantitative Evaluation of Splenic Activity on [^18^F]FDG PET/CT

**Figure 2:**
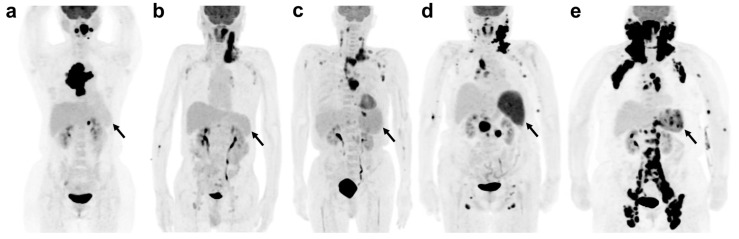
Representative cases of splenic [^18^F]FDG uptake patterns on baseline PET/CT in DLBCL. Maximum intensity projection images from initial staging [^18^F]FDG PET/CT. (**a**) A 40-year-old woman shows negative splenic uptake lower than hepatic uptake (Deauville score 2); the patient remained relapse-free during follow-up. (**b**) A 70-year-old man exhibits positive diffuse splenic uptake higher than the mediastinal blood pool but lower than or equal to the liver (Deauville score 3). (**c**) A 70-year-old man demonstrates positive diffuse splenic uptake moderately higher than hepatic uptake (Deauville score 4). (**d**) An 85-year-old woman shows splenomegaly with positive diffuse splenic uptake markedly exceeding hepatic uptake (Deauville score 5). (**e**) A 67-year-old woman shows positive focal splenic uptake superimposed on diffuse splenic uptake. All four patients with positive splenic uptake patterns (**b**–**e**) experienced disease relapse. Arrows indicate the spleen demonstrating the described uptake pattern.

All [^18^F]FDG PET/CT images were retrospectively reviewed on a dedicated vendor-supplied workstation (Advantage Workstation version 4.7, GE Healthcare, Chicago, IL, USA). A nuclear medicine physician (B.C.), with 16 years of experience, interpreted the [^18^F]FDG PET/CT images while blinded to the clinical outcomes. Initially, visual assessment of splenic uptake was primarily performed using maximum intensity projection images. The degree of uptake was graded according to the Deauville 5-point scale, based on the relative uptake intensity of the mediastinal blood pool and liver [9]. On maximum intensity projection (MIP) images from baseline [^18^F]FDG PET/CT, splenic uptake was visually assessed using a Deauville-based framework. Splenic uptake equal to or lower than that of the mediastinal blood pool was considered negative (Deauville score 1–2). Any splenic uptake exceeding that of the mediastinal blood pool was defined as positive. Specifically, positive splenic uptake patterns were classified as follows: (1) diffuse uptake higher than the mediastinal blood pool but lower than or equal to the liver (Deauville score 3); (2) diffuse uptake moderately increased compared with the liver (Deauville score 4); (3) diffuse uptake markedly increased compared with the liver (Deauville score 5); and (4) focal increased uptake with or without concomitant diffuse splenic uptake. Representative cases for each splenic uptake pattern are shown in Figure 2.

For quantitative analysis, a spherical volume of interest (VOI; 14 cm^3^, 30 mm in diameter) was placed over the area showing the highest [^18^F]FDG uptake within the spleen of each patient. The standardized uptake value maximum and mean, as well as the lean body mass-normalized standardized uptake value maximum and mean, were measured. These quantitative parameters were compared with those obtained from healthy individuals who underwent [^18^F]FDG PET/CT for routine health checkups during the same period.

The spleen size was evaluated on CT images obtained from the PET/CT study, and splenomegaly was determined according to reference values established for the Korean population [17].

### 2.5. Data Analyses

The metabolic parameters of the spleen and clinicopathological factors were compared using the chi-square or Fisher’s exact test for categorical variables. For continuous variables, comparisons were performed using Student’s *t* test or the Mann–Whitney U test for two groups, and one-way ANOVA or Kruskal–Wallis test with post hoc analysis for three or more groups, as appropriate. Clinical variables constituting the IPI and NCCN-IPI, including age, stage, serum LDH, Eastern Cooperative Oncology Group (ECOG) performance status, and the number of extranodal sites, together with [^18^F]FDG PET/CT parameters were evaluated for their association with RFS and OS. Receiver operating characteristic curve analysis was performed to determine the optimal cut-off values for the prediction of RFS and OS, based on the area under the curve. Survival outcomes were analyzed using the Kaplan–Meier method with the log-rank test. Univariate and multivariate analyses were conducted using the Cox proportional hazards model to identify independent predictors of RFS and OS. For each covariate, *p*-values were calculated using the Wald chi-square test for the regression coefficient (β). Variables with *p* < 0.05 in the univariate analysis were entered simultaneously into the multivariate model using the enter method. For both RFS and OS, model discrimination was assessed using Harrell’s C-index. All statistical analyses were performed using MedCalc Statistical Software, version 23.1.7 (MedCalc Software Ltd., Ostend, Belgium; https://www.medcalc.org) and IBM SPSS Statistics for Windows, version 30.0 (IBM Corp., Armonk, NY, USA). Statistical significance was set at *p* < 0.05.

## 3. Results

### 3.1. Patient Characteristics

The baseline characteristics of all 142 patients included are summarized in Table 1. Overall, 43 patients relapsed and 58 died during follow-up. Relapses were associated with advanced Ann Arbor stage and higher IPI-based scores, whereas other baseline variables showed no meaningful differences. In contrast, survival status revealed broader differences, in that deceased patients were older and exhibited more advanced stage, poorer ECOG performance, higher LDH levels, and more frequent extranodal involvement. All prognostic indices correlated strongly with OS, and survival durations were substantially shorter in patients with relapse or death.

### 3.2. Association Between Splenic Uptake on [^18^F]FDG PET/CT and Clinical Outcomes

The associations between splenic [^18^F]FDG uptake and clinical outcomes are presented in Table 2. Positive splenic uptake on [^18^F]FDG PET/CT was observed in 72 of 142 patients. Among 142 patients, 70 (49.3%) showed negative splenic uptake, and 72 (50.7%) showed positive splenic uptake on MIP images. Positive uptake consisted primarily of diffuse uptake patterns (n = 59), which were classified as uptake higher than the mediastinal blood pool but ≤liver (Deauville score 3, n = 42), moderately > liver (Deauville score 4, n = 12), and markedly > liver (Deauville score 5, n = 5). Focal increased uptake was observed in 13 patients. Patients who relapsed showed a significantly higher proportion of positive splenic uptake compared with those who remained relapse-free (*p* < 0.001). Although splenomegaly itself was not significantly associated with relapse or mortality, positive splenic uptake was associated with higher IPI and NCCN-IPI scores in both relapse and survival analyses (all *p* ≤ 0.005). There were no significant differences among all standardized uptake value (SUV) and standardized uptake value normalized to lean body mass parameters according to relapse or death (Supplementary Table S1). When splenic uptake was incorporated into prognostic scoring, the combined IPI and Revised-IPI groupings differed significantly between relapse statuses (*p* = 0.018 and 0.029, respectively), while no risk-group differences were observed for OS.

### 3.3. Survival Outcomes According to Clinical Variables and Splenic [^18^F]FDG Uptake

**Figure 3:**
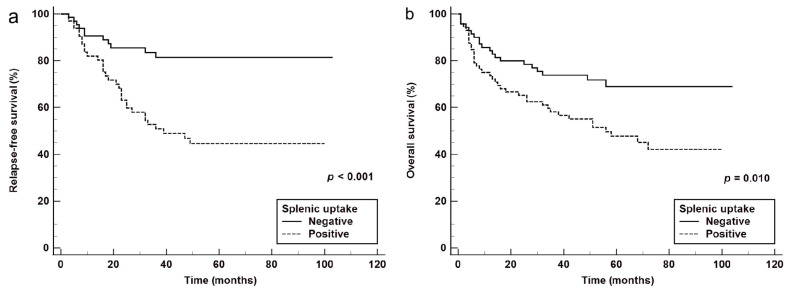
Kaplan–Meier curves for relapse-free survival (**a**) and overall survival (**b**) stratified by splenic uptake on baseline [^18^F]FDG PET/CT. (**a**) Relapse-free survival (RFS) and (**b**) overall survival (OS) according to splenic FDG uptake status. Patients were classified as having negative or positive splenic FDG uptake based on visual interpretation. Patients with positive splenic FDG uptake showed significantly inferior RFS (*p* < 0.001) and OS (*p* = 0.010) compared with those without positive splenic uptake.

Kaplan–Meier survival analyses are presented in Supplementary Table S2, and the corresponding Kaplan–Meier curves stratified by splenic [^18^F]FDG uptake are shown in Figure 3. Because the median survival was not reached, the mean survival times derived from Kaplan–Meier estimates were used to summarize survival outcomes. For RFS, Kaplan–Meier analysis showed significant differences according to Ann Arbor stage (*p* < 0.001), ECOG performance status (*p* = 0.028), extranodal involvement (*p* = 0.027), and splenic [^18^F]FDG uptake (*p* < 0.001). Age categories defined by IPI/R-IPI (*p* = 0.182) and NCCN-IPI (*p* = 0.199) did not demonstrate significant differences in RFS. Similarly, serum LDH levels classified by IPI/R-IPI (*p* = 0.089) and those categorized by NCCN-IPI criteria (*p* = 0.449) were not significant. For OS, significant differences were observed for age based on IPI/R-IPI (*p* = 0.002), NCCN-IPI (*p* = 0.008), serum LDH by IPI/R-IPI (*p* = 0.011), Ann Arbor stage (*p* < 0.001), ECOG performance status (*p* = 0.019), extranodal sites (*p* = 0.007), and splenic uptake (*p* = 0.010). NCCN-IPI LDH categories showed no significant association with OS (*p* = 0.861).

### 3.4. Prognostic Factors Including Splenic Uptake Associated with RFS and OS

In the results of both univariate and multivariate analyses for RFS (Table 3), several prognostic variables demonstrated statistical significance with substantial effect sizes. In univariate analysis, advanced Ann Arbor stage (III–IV) and positive splenic uptake emerged as robust predictors, representing a four-fold (HR: 4.024, *p* < 0.001) and three-fold (HR: 3.470, *p* < 0.001) increase in the risk of relapse, respectively. Poor ECOG performance status (HR: 2.529, *p* = 0.035) and ≥2 extranodal sites (HR: 1.977, *p* = 0.031) were also significant risk factors. In the multivariate model, positive splenic uptake remained a significant independent prognostic factor, conferring a more than two-fold increased risk of relapse (HR: 2.175, *p* = 0.043), alongside advanced stage which maintained a high hazard ratio (HR: 2.872, *p* = 0.004).

Table 4 summarizes the univariate and multivariate analyses of factors associated with OS. In univariate analysis, positive splenic uptake was associated with a two-fold increase in the risk of mortality (HR: 1.996, *p* = 0.012). Other significant factors associated with inferior OS included older age (>60 years; HR: 2.860, *p* = 0.004), advanced Ann Arbor stage (III–IV; HR: 3.520, *p* < 0.001), poor ECOG performance status (HR: 2.279, *p* = 0.023), elevated LDH (HR: 1.994, *p* = 0.014), and ≥2 extranodal sites (HR: 2.012, *p* = 0.009). In the multivariate model, age > 60 years (HR: 2.501, *p* = 0.012) and advanced Ann Arbor stage III–IV (HR: 3.253, *p* < 0.001) remained the dominant independent predictors, suggesting that the prognostic contribution of splenic uptake for OS may be attenuated after accounting for these key clinical factors. Similarly, in the NCCN-IPI model, age ≥ 75 years (HR: 8.400, *p* = 0.038) and advanced Ann Arbor stage (HR: 3.374, *p* < 0.001) remained independently associated with inferior OS.

### 3.5. Prognostic Discrimination of Clinical and Combined Splenic Uptake Models

Harrell’s C-index analysis for RFS showed values of 0.661 (95% confidence interval [CI]: 0.589–0.733) for the IPI group, 0.660 (95% CI: 0.591–0.730) for the R-IPI group, and 0.671 (95% CI: 0.599–0.743) for the NCCN-IPI group. Models incorporating splenic [^18^F]FDG uptake demonstrated C-indices of 0.690 (95% CI: 0.619–0.762) for the IPI plus splenic-positive group, 0.642 (95% CI: 0.573–0.711) for the R-IPI plus splenic-positive group, and 0.680 (95% CI: 0.605–0.754) for the NCCN-IPI plus splenic-positive group. For OS, the C-indices were 0.695 (95% CI: 0.631–0.759) for IPI, 0.687 (95% CI: 0.631–0.742) for R-IPI, and 0.703 (95% CI: 0.647–0.759) for NCCN-IPI. The corresponding splenic-positive combined models showed C-indices of 0.688 (95% CI: 0.623–0.752), 0.678 (95% CI: 0.624–0.731), and 0.690 (95% CI: 0.631–0.750), respectively (Supplementary Table S3).

## 4. Discussion

Splenic metabolic activity on baseline [^18^F]FDG PET/CT demonstrated meaningful prognostic relevance in patients with DLBCL. Qualitative assessment using a Deauville-based visual scale identified a substantial proportion of patients with an abnormally increased splenic uptake, which showed significant associations with adverse clinical features, including higher IPI and NCCN-IPI scores. Positive splenic uptake not only correlated with increased relapse rates and inferior survival outcomes, but also remained an independent predictor of RFS after adjustment for conventional clinical variables. When incorporated into existing prognostic indices, splenic uptake modestly enhanced the discrimination of selected models for RFS, although improvements were not observed across all indices or for OS. These findings suggest that splenic metabolic activity reflects clinically significant disease biology and may serve as an easily assessed imaging biomarker that complements conventional risk stratification systems in newly diagnosed DLBCL.

The prognostic significance of the spleen underscores its central role as a secondary lymphoid organ involved in immune activation, lymphocyte trafficking, and cytokine regulation. Increased metabolic activity within the spleen may reflect systemic inflammation, subclinical dissemination, or heightened tumor–host immune interaction, all of which are relevant in aggressive lymphomas. Prior imaging studies have demonstrated that splenic abnormalities on CT, magnetic resonance imaging, or PET/CT frequently accompany hematologic malignancies and may influence staging or treatment decisions [14,18]. Furthermore, previous PET-based investigations have shown that diffuse or focal splenic FDG uptake can serve as a marker of systemic disease burden in lymphoma, potentially aiding in prognosis and treatment planning [15,19,20]. In our cohort, positive splenic uptake correlated strongly with relapses and high-risk clinical scores, supporting these prior observations and suggesting that splenic metabolic activity captures clinically significant disease biology that is not accounted for by conventional staging alone.

Several PET/CT-based parameters, including total metabolic tumor volume, total lesion glycolysis, and SUV-based indices, have been reported as robust prognostic markers in DLBCL [10,21,22,23,24,25]. These metrics quantify global metabolic tumor burden and often require intensive manual segmentation or specialized software, limiting routine clinical integration. In contrast, in the current study, splenic metabolic assessment was simple, rapidly identifiable on maximum intensity projection images, and reproducible across readers. Although it does not replace volumetric tumor-burden metrics, splenic activity represents a distinct biologic PET feature reflecting organ-specific immune or metabolic activation and may complement established PET/CT-derived prognostic parameters.

RFS has historically been effectively stratified using the IPI, R-IPI, and NCCN-IPI, with large international cohorts consistently demonstrating meaningful separation between risk groups [4,5,6,26]. In our study, the prognostic performance of these indices fell within similar ranges but demonstrated reduced separation between risk categories. The older age distribution and high prevalence of advanced-stage disease in our cohort may have compressed the prognostic gradients, diminishing the distinction among clinical subgroups. Additionally, the modest number of relapse events may have limited statistical power. Indeed, our observed C-indices (0.661 for IPI, 0.660 for R-IPI, and 0.671 for NCCN-IPI) are consistent with external validation studies reporting modest discrimination for progression-free outcomes (e.g., 2-year PFS C-indices of 0.610, 0.600, and 0.622, respectively) [27]. Nevertheless, the incorporation of splenic uptake improved discrimination in selected RFS models, suggesting that metabolic imaging biomarkers can strengthen prognostic performance when baseline clinical risk stratification is attenuated by population characteristics.

For OS, our findings were consistent with those of prior studies demonstrating strong prognostic performance of the IPI, R-IPI, and particularly the NCCN-IPI [7,26,28,29]. In our cohort, the observed C-indices were 0.695 for the IPI, 0.687 for the R-IPI, and 0.703 for the NCCN-IPI, which are comparable to those reported in a large pooled analysis (e.g., 0.626, 0.590, and 0.632, respectively) [7]. However, adding splenic uptake did not improve OS prediction. This may reflect the high proportion of elderly patients, in whom competing non-lymphoma mortality influences survival outcomes [30,31]. Because OS captures long-term comorbidities, treatment-related complications, and non-cancer causes of death, the prognostic influence of lymphoma-specific metabolic parameters may be diluted. These factors likely explain the limited incremental value of splenic uptake in OS prediction despite its relevance for RFS.

This study has several limitations. First, it was conducted in a single-center cohort of Korean patients, which may limit generalizability and introduce selection bias. Second, non-relapse patients exhibited shorter OS follow-up than relapse patients, partly due to the older age distribution and earlier administrative censoring; this imbalance may influence long-term survival estimation. Third, treatment-related factors such as regimen selection, dose intensity, and treatment modifications were not incorporated, allowing potential residual confounding. Fourth, although splenic uptake was assessed using a standardized qualitative method, physiologic variability in spleen metabolism may affect binary classification. Additionally, this study focused exclusively on baseline PET/CT to avoid the confounding effects of chemotherapy-induced inflammatory or immune-related changes in splenic metabolism; therefore, dynamic changes in splenic uptake during treatment were not evaluated. Finally, the modest sample size reduced the statistical power and precluded detailed subgroup analyses. Larger, prospective multicenter studies using standardized PET/CT protocols are necessary to validate these findings.

## 5. Conclusions

Splenic metabolic activity on baseline [^18^F]FDG PET/CT was strongly associated with relapse and adverse clinical features in this cohort of Korean patients with DLBCL. Splenic uptake provided incremental prognostic value for RFS when incorporated into established clinical indices, although its contribution to OS prediction was limited. Splenic activity is easily assessed, reproducible, and biologically relevant, and thus may serve as a useful adjunctive imaging biomarker in baseline prognostic evaluation. Further prospective studies are warranted to refine the role of spleen-directed metabolic assessment and determine how best to integrate it with advanced quantitative PET parameters and emerging molecular risk classifiers.

## Figures and Tables

**Table 1:** Baseline characteristics of patients with DLBCL according to relapse and survival status.

		Relapse			Overall Survival		
	Overall	Relapse-Free	Relapsed	*p* Value	Alive	Deceased	*p* Value
Patients (n)	142	99	43		84	58	
Male/Female	80/62	57/42	23/20	0.652	46/38	34/24	0.649
Age	65.8 ± 13.6	65.5 ± 14.1	66.5 ± 12.5	0.665	62.5 ± 13.6	70.5 ± 12.1	<0.001
Age (≤60/>60 years)	41/101	30/69	11/32	0.568	32/52	9/49	0.004
Age (≤40, 41–60, 61–75, >75 years)	9/32/67/34	8/22/42/27	1/10/25/7	0.226	8/24/38/14	1/8/29/20	0.010
Ann Arbor Stage (I/II/III/IV)	25/48/26/43	19/41/14/25	6/7/12/18	0.008	18/38/10/18	7/10/16/25	<0.001
Ann Arbor Stage (I–II/III–IV)	73/69	60/39	13/30	<0.001	56/28	17/41	<0.001
ECOG performance status (0/1/2/3)	38/92/8/4	30/63/4/2	8/29/4/2	0.238	34/47/1/2	4/45/7/2	<0.001
ECOG performance status (0–1/2–3)	130/12	93/6	37/6	0.185	81/3	49/9	0.015
Serum LDH (U/L)	359.0 ± 310.7	325.7 ± 196.8	435.6 ± 474.3	0.149	293.7 ± 147.3	453.5 ± 437.9	0.009
LDH (≤250/>250)	66/76	50/49	16/27	0.144	47/37	19/39	0.006
LDH (≤250/251–750/>750)	65/66/11	44/45/10	21/21/1	0.320	38/40/6	27/26/5	0.901
Extranodal sites (0–1/≥2)	99/43	72/27	27/16	0.236	65/19	34/24	0.017
Relapse-free survival (months)	38.2 ± 29.3	46.8 ± 30.4	18.3 ± 12.2	<0.001	46.8 ± 30.4	38.1 ± 26.6	0.106
Overall survival (months)	44.2 ± 29.5	54.9 ± 25.1	14.0 ± 13.9	<0.001	61.6 ± 21.8	19.0 ± 19.1	<0.001
IPI score	2.1 ± 1.4	1.9 ± 1.4	2.6 ± 1.4	0.007	1.7 ± 1.3	2.8 ± 1.3	<0.001
NCCN-IPI score	3.4 ± 1.6	3.2 ± 1.7	3.8 ± 1.5	0.041	2.8 ± 1.5	4.2 ± 1.4	<0.001
IPI group	58/23/30/31	46/17/19/17	12/6/11/14	0.088	46/16/9/13	12/7/21/18	<0.001
Revised-IPI group	19/62/61	16/47/36	3/15/25	0.043	17/45/22	2/17/39	<0.001
NCCN-IPI group	20/58/52/12	16/45/30/8	4/13/22/4	0.104	17/45/20/2	3/13/32/10	<0.001

All patients included in this study were of East Asian ethnicity (Korean). Data are expressed as mean ± standard deviation or number of patients. *p* values were obtained using the χ^2^ test, Fisher’s exact test, independent *t* test, or Mann–Whitney U test, as appropriate. LDH, lactate dehydrogenase; ECOG, Eastern Cooperative Oncology Group; IPI, International Prognostic Index; NCCN, National Comprehensive Cancer Network.

**Table 2:** Association between splenic uptake on [^18^F]FDG PET/CT and clinical outcomes in patients with DLBCL.

		Relapse			Overall Survival		
	Overall	Relapse-Free (n = 99)	Relapsed (n = 43)	*p* Value	Alive (n = 84)	Deceased (n = 58)	*p* Value
Splenic uptake (negative/positive)	70/72	59/40	11/32	<0.001	50/34	20/38	0.003
Splenomegaly (no/yes)	115/27	84/15	31/12	0.075	72/12	43/15	0.084
IPI score + SpU (+)	2.6 ± 1.7	2.3 ± 1.6	3.4 ± 1.9	<0.001	2.1 ± 1.6	3.5 ± 1.5	<0.001
NCCN-IPI score + SpU (+)	3.9 ± 1.9	3.6 ± 1.9	4.5 ± 1.8	0.005	3.2 ± 1.7	4.9 ± 1.6	<0.001
IPI group + SpU (+)	47/22/26/47	30/11/18/40	17/11/8/7	0.018	34/11/15/24	13/11/11/23	0.142
Revised-IPI group + SpU (+)	14/55/73	9/32/58	5/23/15	0.029	9/36/39	5/19/34	0.359
NCCN-IPI group + SpU (+)	15/51/43/33	10/30/32/27	5/21/11/6	0.130	10/34/22/18	5/17/21/15	0.397

Data are expressed as mean ± standard deviation or number of patients. For score and risk-group variables combined with splenic uptake, 1 point was added to the respective prognostic index for patients with positive splenic uptake on [^18^F]FDG PET/CT. *p* values were obtained using the χ^2^ test, Fisher’s exact test, independent *t* test, or Mann–Whitney U test, as appropriate. SpU (+), splenic uptake-positive; IPI, International Prognostic Index; NCCN, National Comprehensive Cancer Network.

**Table 3:** Results of univariate and multivariate analyses for prediction of relapse-free survival.

	Univariate Analysis			Multivariate Analysis		
Variables	HR	95% CI	*p* Value	HR	95% CI	*p* Value
IPI Age (≤60/>60 years)	1.583	0.797–3.145	0.189			
IPI Ann Arbor Stage (I–II/III–IV)	4.024	2.090–7.749	<0.001 *	2.872	1.398–5.897	0.004 *
IPI ECOG performance status (0–1/2–3)	2.529	1.066–6.002	0.035 *			0.498
IPI LDH (≤250/>250 U/L)	1.693	0.912–3.114	0.095			
IPI EN (0–1/≥2)	1.977	1.063–3.676	0.031 *			0.332
Splenic uptake (negative/positive)	3.470	1.748–6.890	<0.001 *	2.175	1.024–4.620	0.043 *
NCCN-IPI Age (≤40 years)			0.250			
NCCN-IPI Age (41–60 years)	3.425	0.438–26.761	0.241			
NCCN-IPI Age (61–75 years)	5.097	0.690–37.642	0.110			
NCCN-IPI Age (≥75 years)	3.048	0.375–24.804	0.297			
NCCN-IPI Ann Arbor Stage (I–II/III–IV)	4.024	2.090–7.749	<0.001 *	2.872	1.398–5.897	0.004 *
NCCN-IPI ECOG performance status (0–1/2–3)	2.529	1.066–6.002	0.035 *			0.498
NCCN-IPI Serum LDH (≤250 U/L)			0.495			
NCCN-IPI LDH (251–750 U/L)	1.021	0.557–1.869	0.947			
NCCN-IPI LDH (>750 U/L)	0.305	0.041–2.268	0.246			
NCCN-IPI EN (0–1/≥2)	1.977	1.063–3.676	0.031 *			0.332
Splenic uptake (negative/positive)	3.470	1.748–6.890	<0.001 *	2.175	1.024–4.620	0.043 *

* Statistically significant. IPI, International Prognostic Index; NCCN, National Comprehensive Cancer Network; ECOG, Eastern Cooperative Oncology Group; LDH, lactate dehydrogenase; EN, extranodal sites.

**Table 4:** Results of univariate and multivariate analyses for prediction of overall survival.

Variables	Univariate Analysis			Multivariate Analysis		
	HR	95% CI	*p* Value	HR	95% CI	*p* Value
IPI Age (≤60/>60 years)	2.860	1.402–5.835	0.004 *	2.501	1.223–5.115	0.012 *
IPI Ann Arbor Stage (I–II/III–IV)	3.520	1.993–6.216	<0.001 *	3.253	1.838–5.757	<0.001 *
IPI ECOG performance status (0–1/2–3)	2.279	1.119–4.643	0.023 *			0.190
IPI LDH (≤250/>250 U/L)	1.994	1.151–3.454	0.014 *			0.931
IPI EN (0–1/≥2)	2.012	1.191–3.399	0.009 *			0.489
Splenic uptake (negative/positive)	1.996	1.161–3.432	0.012 *			0.470
NCCN-IPI Age (≤40 years)			0.016 *			0.033 *
NCCN-IPI Age (41–60 years)	2.499	0.313–19.988	0.388	3.176	0.400–27.049	0.277
NCCN-IPI Age (61–75 years)	5.335	0.726–39.205	0.100	5.479	0.745–40.305	0.095
NCCN-IPI Age (≥75 years)	7.843	1.051–58.542	0.045 *	8.400	1.124–62.785	0.038 *
NCCN-IPI Ann Arbor Stage (I–II/III–IV)	3.520	1.993–6.216	<0.001 *	3.374	1.902–5.987	<0.001 *
NCCN-IPI ECOG performance status (0–1/2–3)	2.279	1.119–4.643	0.023 *			0.083
NCCN-IPI Serum LDH (≤250 U/L)			0.862			
NCCN-IPI LDH (251–750 U/L)	0.924	0.539–1.584	0.692			
NCCN-IPI LDH (>750 U/L)	1.305	0.459–3.099	0.643			
NCCN-IPI EN (0–1/≥2)	2.012	1.191–3.399	0.009 *			0.550
Splenic uptake (negative/positive)	1.996	1.161–3.432	0.012 *			0.470

* Statistically significant. IPI, International Prognostic Index; NCCN, National Comprehensive Cancer Network; ECOG, Eastern Cooperative Oncology Group; LDH, lactate dehydrogenase; EN, extranodal sites.

## Data Availability

The data used in this study are not publicly available due to patient privacy concerns and institutional restrictions but are available from the corresponding author upon reasonable request.

## Supplementary Materials

The following supporting information can be downloaded at: https://www.mdpi.com/article/10.3390/cancers18030449/s1, Table S1: Comparison of splenic [^18^F]FDG PET/CT parameters in patients with DLBCL. Table S2: Kaplan–Meier analysis of relapse-free and overall survival according to clinical and [^18^F]FDG PET/CT parameters in patients with DLBCL. Table S3: Harrell’s C-Indices for predicting RFS and OS using clinical indices and splenic [^18^F]FDG uptake in patients with DLBCL.

## Abbreviations

The following abbreviations are used in this manuscript:

DLBCL	Diffuse large B-cell lymphoma
NHL	Non-Hodgkin lymphoma
IPI	International Prognostic Index
R-IPI	Revised International Prognostic Index
NCCN-IPI	National Comprehensive Cancer Network–International Prognostic Index
LDH	Lactate dehydrogenase
[^18^F]FDG	Fluorine-18 fluorodeoxyglucose
PET/CT	Positron emission tomography/computed tomography
FDG	Fluorodeoxyglucose
RFS	Relapse-free survival
OS	Overall survival
CT	Computed tomography
VOI	Volume of interest
SUV	Standardized uptake value
HR	Hazard ratio
CI	Confidence interval
ECOG	Eastern Cooperative Oncology Group

## References

[1] Siegel R.L., Kratzer T.B., Giaquinto A.N., Sung H., Jemal A. (2025). Cancer statistics, 2025. CA Cancer J. Clin..

[2] Wang S.S. (2023). Epidemiology and etiology of diffuse large B-cell lymphoma. Semin. Hematol..

[3] Kim J.S., Liu Y., Ha K.H., Qiu H., Rothwell L.A., Kim H.C. (2020). Increasing incidence of B-cell non-Hodgkin lymphoma and occurrence of second primary malignancies in South Korea: 10-year follow-up using the Korean National Health Information database. Cancer Res. Treat..

[4] (1993). International Non-Hodgkin’s Lymphoma Prognostic Factors Project. A predictive model for aggressive non-Hodgkin’s lymphoma. N. Engl. J. Med..

[5] Sehn L.H., Berry B., Chhanabhai M., Fitzgerald C., Gill K., Hoskins P., Klasa R., Savage K.J., Shenkier T., Sutherland J. (2007). The revised International Prognostic Index (R-IPI) is a better predictor of outcome than the standard IPI for patients with diffuse large B-cell lymphoma treated with R-CHOP. Blood.

[6] Zhou Z., Sehn L.H., Rademaker A.W., Gordon L.I., Lacasce A.S., Crosby-Thompson A., Vanderplas A., Zelenetz A.D., Abel G.A., Rodriguez M.A. (2014). An enhanced International Prognostic Index (NCCN-IPI) for patients with diffuse large B-cell lymphoma treated in the rituximab era. Blood.

[7] Ruppert A.S., Dixon J.G., Salles G., Wall A., Cunningham D., Poeschel V., Haioun C., Tilly H., Ghesquieres H., Ziepert M. (2020). International prognostic indices in diffuse large B-cell lymphoma: A comparison of IPI, R-IPI, and NCCN-IPI. Blood.

[8] Cheson B.D., Fisher R.I., Barrington S.F., Cavalli F., Schwartz L.H., Zucca E., Lister T.A. (2014). Recommendations for initial evaluation, staging, and response assessment of Hodgkin and non-Hodgkin lymphoma: The Lugano classification. J. Clin. Oncol..

[9] Barrington S.F., Mikhaeel N.G., Kostakoglu L., Meignan M., Hutchings M., Müeller S.P., Schwartz L.H., Zucca E., Fisher R.I., Trotman J. (2014). Role of imaging in the staging and response assessment of lymphoma: Consensus of the International Conference on Malignant Lymphomas Imaging Working Group. J. Clin. Oncol..

[10] Vercellino L., Cottereau A.-S., Casasnovas O., Tilly H., Feugier P., Chartier L., Fruchart C., Roulin L., Oberic L., Pica G.M. (2020). High total metabolic tumor volume at baseline predicts survival independent of response to therapy. Blood.

[11] Cottereau A.S., Meignan M., Nioche C., Capobianco N., Clerc J., Chartier L., Vercellino L., Casasnovas O., Thieblemont C., Buvat I. (2021). Risk stratification in diffuse large B-cell lymphoma using lesion dissemination and metabolic tumor burden calculated from baseline PET/CT^†^. Ann. Oncol..

[12] Kostakoglu L., Mattiello F., Martelli M., Sehn L.H., Belada D., Ghiggi C., Chua N., González-Barca E., Hong X., Pinto A. (2022). Total metabolic tumor volume as a survival predictor for patients with diffuse large B-cell lymphoma in the GOYA study. Haematologica.

[13] Lewis S.M., Williams A., Eisenbarth S.C. (2019). Structure and function of the immune system in the spleen. Sci. Immunol..

[14] Saboo S.S., Krajewski K.M., O’Regan K.N., Giardino A., Brown J.R., Ramaiya N., Jagannathan J.P. (2012). Spleen in haematological malignancies: Spectrum of imaging findings. Br. J. Radiol..

[15] Wang S., Ju H., Bai Y., Wang L., Ding Q., Li P., Jiang X., Lin X. (2023). The prognostic value of splenic abnormalities in pretreatment ^18^F-FDG PET/CT in patients with complete response diffuse large B-cell lymphoma. Clin. Radiol..

[16] Gerards N.R., Wiegers S.E., Bes A.L., Eertink J.J., Lugtenburg P.J., Zijlstra J.M., Boellaard R., Zwezerijnen G.J.C. (2025). Can we obtain prognostic information from healthy tissue uptake and volume in baseline ^18^F-FDG PET/CT imaging in diffuse large B-cell lymphoma?. Eur. J. Nucl. Med. Mol. Imaging.

[17] Lee H.A., Kim S.U., Lim J., Kim M.Y., Kim S.G., Suk K.T., Jang J.Y., An H., Yim H.J., Seo Y.S. (2023). Age, sex, and body mass index should be considered when assessing spleen length in patients with compensated advanced chronic liver disease. Gut Liver.

[18] Rini J.N., Leonidas J.C., Tomas M.B., Palestro C.J. (2003). ^18^F-FDG PET versus CT for evaluating the spleen during initial staging of lymphoma. J. Nucl. Med..

[19] Zhao Z., Zhou Y., Yao X., Ge S., Sang S., Yang Y., Zhang B., Deng S. (2024). Prognostic significance of diffuse increased fluorine-18-fluorodeoxyglucose (^18^F-FDG) uptake within the reticuloendothelial system in lymphoma patients. Quant. Imaging Med. Surg..

[20] Liu Y. (2009). Clinical significance of diffusely increased splenic uptake on FDG-PET. Nucl. Med. Commun..

[21] Sasanelli M., Meignan M., Haioun C., Berriolo-Riedinger A., Casasnovas R.O., Biggi A., Gallamini A., Siegel B.A., Cashen A.F., Véra P. (2014). Pretherapy metabolic tumour volume is an independent predictor of outcome in patients with diffuse large B-cell lymphoma. Eur. J. Nucl. Med. Mol. Imaging.

[22] Song M.K., Yang D.H., Lee G.W., Lim S.N., Shin S., Pak K.J., Kwon S.Y., Shim H.K., Choi B.H., Kim I.S. (2016). High total metabolic tumor volume in PET/CT predicts worse prognosis in diffuse large B cell lymphoma patients with bone marrow involvement in rituximab era. Leuk. Res..

[23] Ilyas H., Mikhaeel N.G., Dunn J.T., Rahman F., Møller H., Smith D., Barrington S.F. (2018). Defining the optimal method for measuring baseline metabolic tumour volume in diffuse large B cell lymphoma. Eur. J. Nucl. Med. Mol. Imaging.

[24] Terasawa T., Lau J., Bardet S., Couturier O., Hotta T., Hutchings M., Nihashi T., Nagai H. (2009). Fluorine-18-fluorodeoxyglucose positron emission tomography for interim response assessment of advanced-stage Hodgkin’s lymphoma and diffuse large B-cell lymphoma: A systematic review. J. Clin. Oncol..

[25] Paes F.M., Kalkanis D.G., Sideras P.A., Serafini A.N. (2010). FDG PET/CT of extranodal involvement in non-Hodgkin lymphoma and Hodgkin disease. RadioGraphics.

[26] Montalbán C., Díaz-López A., Dlouhy I., Rovira J., Lopez-Guillermo A., Alonso S., Martín A., Sancho J.M., García O., Sánchez J.M. (2017). Validation of the NCCN-IPI for diffuse large B-cell lymphoma (DLBCL): The addition of β2 -microglobulin yields a more accurate GELTAMO-IPI. Br. J. Haematol..

[27] Warnnissorn N., Kanitsap N., Niparuck P., Boonsakan P., Kulalert P., Limvorapitak W., Bhoopat L., Saengboon S., Chantrathammachart P., Puavilai T. (2022). External validation and comparison of IPI, R-IPI, and NCCN-IPI in diffuse large B-cell lymphoma patients treated with R-CHOP to predict 2-year progression-free survival. Hematology.

[28] Huang C.-E., Chen Y.-Y., Lu C.-H., Chen P.-T., Lee K.-D., Chen C.-C. (2015). Validation of an enhanced International Prognostic Index (NCCN-IPI) in an Asian cohort of patients with diffuse large B cell lymphoma. Ann. Hematol..

[29] Salas M.Q., Mercadal S., Domingo Domenech E., Oliveira A.C., Encuentra M., Climent F., Andrade Campos M., Aguilera C., Fernández de Sevilla A., Sureda A. (2020). Validation of the NCCN-IPI and the GELTAMO-IPI for diffuse large B-cell lymphoma treated with R-CHOP in a large cohort of patients from a single institution. Leuk. Lymphoma.

[30] Barrington S.F., Zwezerijnen B.G.J.C., de Vet H.C.W., Heymans M.W., Mikhaeel N.G., Burggraaff C.N., Eertink J.J., Pike L.C., Hoekstra O.S., Zijlstra J.M. (2021). Automated segmentation of baseline metabolic total tumor burden in diffuse large B-cell lymphoma: Which method is most successful? A study on behalf of the PETRA consortium. J. Nucl. Med..

[31] Byun J.M., Lee J.O., Kang B., Kim J.W., Kim S.H., Kim J.W., Kim Y.J., Lee K.W., Bang S.M., Lee J.S. (2016). Diffuse large B-cell lymphoma in the elderly: Real world outcomes of immunochemotherapy in Asian population. Clin. Lymphoma Myeloma Leuk..

