# Supplementary material for: Prognostic Value of Qualitative Splenic [18F]FDG Uptake on Baseline PET/CT in Newly Diagnosed Diffuse Large B-Cell Lymphoma
Source: Cancers (Basel). 2026 Jan 30;18(3):449. doi: 10.3390/cancers18030449 (PMC12896426; doi:10.3390/cancers18030449)
Supplement: Supplementary file 1 [file cancers-18-00449-s001.zip › cancers-4082483-supplementary.pdf]

Table S1. Comparison of splenic [^{18}F]FDG PET/CT parameters in patients with DLBCL.

		Relapse			Overall survival		
	Overall	Relapse-free	Relapsed	*p* value	Alive	Deceased	*p* value
Spleen SUVmax	3.62±3.90	3.68±4.04	4.95±5.26	0.164	4.20±5.01	3.88±3.57	0.683
Spleen SUVpeak	3.10±3.09	3.21±3.44	4.10±3.70	0.166	3.57±3.91	3.35±2.92	0.716
Spleen SULmax	2.79±2.96	2.84±3.08	3.84±3.93	0.145	3.23±3.76	3.02±2.76	0.720
Spleen SULpeak	2.39±2.38	2.48±2.66	3.19±2.81	0.154	2.75±2.98	2.61±2.31	0.768

Table S2. Kaplan–Meier analysis of relapse-free and overall survival according to clinical and [18F]FDG PET/CT parameters in patients with DLBCL.

	Relapse-free survival			Overall survival		
Variable	Mean survival times (months)	95% CI	*p* value	Mean survival times (months)	95% CI	*p* value
IPI and R-IPI Age (≤ 60 / > 60 years)			0.182			0.002*
Age (≤ 60 years)	78.9	66.9–91.0		84.7	74.1–95.4	
Age (> 60 years)	64.9	56.1–73.6		59.6	50.7–68.5	
NCCN-IPI Age			0.199			0.008*
Age (40 ≤ years)	84.5	72.6–96.4		81.2	74.7–95.6	
Age (41–60 years)	74.2	59.7–88.8		81.7	68.8–94.6	
Age (61–75 years)	61.2	50.5–71.8		63.9	52.9–74.9	
Age (75 ≥ years)	66.0	53.5–78.6		45.1	33.1–57.0	
IPI and R-IPI serum LDH (U/L)			0.089			0.011*
LDH (250 ≤)	78.7	68.6–88.8		77.4	67.2–87.5	
LDH (250 >)	59.5	50.2–68.9		55.6	46.2–65.0	
NCCN-IPI Serum LDH (U/L)			0.449			0.861
LDH (250 ≤)	70.5	59.5–81.5		66.2	55.3–77.1	
LDH (251–750)	69.8	58.7–80.8		67.4	57.0–77.8	
LDH (> 750)	77.0	60.7–93.3		52.3	29.6–74.9	
Ann Arbor Stage	71.8	64.3–79.3	<0.001*			<0.001*
Stage (I–II)	86.6	78.5–94.6		83.0	74.6–91.4	
Stage (III–IV)	45.9	36.2–55.6		48.9	38.2–59.6	
ECOG performance status			0.028*			0.019*
ECOG performance status (0–1)	74.0	66.3–81.6		69.7	62.1–77.3	
ECOG performance status (2–3)	37.6	17.4–57.8		36.8	19.8–53.7	
Extranodal sites			0.027*			0.007*
Extranodal sites (0–1)	76.6	68.3–84.9		73.6	65.2–81.9	
Extranodal sites (≥ 2)	50.4	37.6–63.2		49.3	36.3–62.2	
Splenic uptake on [18F]FDG PET/CT			<0.001*			0.010*
Splenic uptake (negative)	86.8	78.1–95.5		77.5	67.7–87.3	
Splenic uptake (positive)	56.0	45.5–66.5		56.1	46.3–65.9	

Mean survival times (months) were estimated from Kaplan–Meier survival curves because median survival was not reached in most subgroups. *p* values were calculated using the log-rank test. DLBCL, diffuse large B-cell lymphoma; IPI, International Prognostic Index; R-IPI, Revised International Prognostic Index; NCCN-IPI, National Comprehensive Cancer Network-International Prognostic Index; LDH, lactate dehydrogenase; ECOG, Eastern Cooperative Oncology Group; RFS, relapse-free survival; OS, overall survival.

Table S3. Harrell's C-Indices for predicting RFS and OS using clinical indices and splenic [^{18}F]FDG uptake in patients with DLBCL.

		Harrell's C-index	95% Confidence interval	p value
RFS	IPI group	0.661	0.589–0.733	0.0035
	R-IPI group	0.660	0.591–0.730	0.0002
	NCCN-IPI group	0.671	0.599–0.743	0.0021
	IPI group + splenic uptake-positive	0.690	0.619–0.762	0.0002
	Revised-IPI group + splenic uptake-positive	0.642	0.573–0.711	0.0030
	NCCN-IPI group + splenic uptake-positive	0.680	0.605–0.754	0.0003
OS	IPI	0.695	0.631–0.759	<0.0001
	R-IPI	0.687	0.631–0.742	<0.0001
	NCCN-IPI	0.703	0.647–0.759	<0.0001
	IPI group + splenic uptake-positive	0.688	0.623–0.752	<0.0001
	Revised-IPI group + splenic uptake-positive	0.678	0.624–0.731	<0.0001
	NCCN-IPI group + splenic uptake-positive	0.690	0.631–0.750	<0.0001

IPI, International Prognostic Index; NCCN, National Comprehensive Cancer Network.
